# Deep vein thrombosis as a rare complication of brucellosis

**Published:** 2014

**Authors:** Ali Reza Davoudi, Atefe Tayebi, Narges Najafi, Elnaz Kasiri

**Affiliations:** 1 Department of Infectious Disease, Antimicrobial Resistance Center, Mazandaran University of Medical Sciences, Sari, Iran; 2 Faculty of Medicine, Mazandaran University of Medical Sciences, Sari, Iran

**Keywords:** Deep vein thrombosis, Thrombophlebitis, Brucellosis

## Abstract

***Background: ***Brucellosis can involve almost any organ system and may present with a broad spectrum of clinical presentations. In this study, we present a case of deep vein thrombosis due to human brucellosis.

***Case Presentation:*** A 15- year old boy presented with acute pain and swelling in his left thigh in June 2011, when he complained of fever, chills and lower extremity pain in which he could barely walk. In family history, his older brother had brucellosis 3 weeks ago and appropriate medication was given. The tubal standard agglutination test (wright test) and 2ME test were positive (in a titer of 1/1280 and 1/640, respectively). Peripheral venous doppler ultrasound of left lower extremity showed that common iliac, femoral, external iliac, superficial and deep femoral vein and popliteal vein were enlarged and contained with echogenous clot. He was treated with rifampicin 600 mg once a day, doxycycline 100 mg twice a day (both for three months) and amikacin 500 mg twice a day (for 2 weeks) accompanied with anti-coagulant. Ten days after the onset of this treatment, thrombophlebitis was cured. The follow up of the patient showed no abnormality after approximately one year later.

***Conclusion: ***In brucellosis endemic areas, the clinicians who encounter patients with deep vein thrombosis and current history of a febrile illness, should consider the likelihood of brucellosis.

Brucellosis is a systemic infection caused by intracellular bacteria of the genus brucella which is transmitted from animal to human. A multisystem infection can involve almost any organ system and may present with a broad spectrum of clinical presentations (1, 2). Although osteoarticular complication is common in the endemic region of brucellosis, but vascular complication is rarely reported in the endemic regions (3, 4). In this paper, we present a case of deep vein thrombosis in Sari, North of Iran. 

## Case Presentation

A 15- year old boy presented to a local general clinic with acute pain and swelling in his left thigh on June 11, 2011. His symptoms started with fever, chills, night sweating and malaise one month earlier. Ten days before admission, he was suffering from pain in his left leg and was unable to walk easily. First he was visited by a general practitioner who asked for some lab tests then referred him to our hospital (Ghaemshahr Razi Hospital) for further evaluation. When admitted to the hospital, he complained of fever, chills and lower extremity pain in which he could barely walk. He had no nausea, vomiting, diarrhea or coryza sign. He was a shephered and had frequent exposures with farm animals and a history of consumption of unpasteurized milk products. 

In family history, his older brother had been diagnosed with brucellosis 3 weeks ago and appropriate medication was given. On admission the patient appeared ill, his temperature was 38.5ºC, blood pressure was 100/60 mmHg and heart rate was 105 beats/min. 

In extremities examination, the left lower extremity enlarged (30 cm), warm, slightly erythematous and tender. The diameter of right lower extremity was 23 cm. Examination of the other organs was normal. Complete blood count and biochemical parameters were within normal range. Erythrocyte sedimentation rate (ESR) was 28 mm/h. The D-dimer test was positive (6410.93 mg/L with normal value of <500 mg/L). 

**Figure 1:**
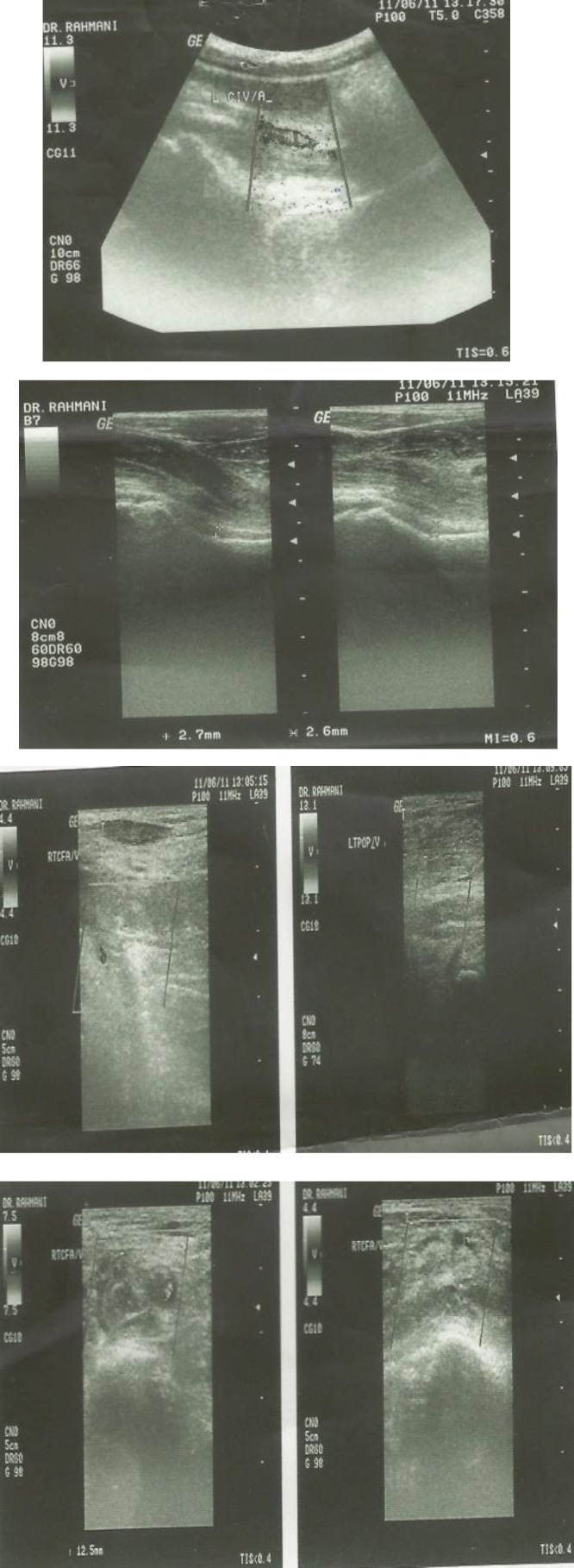
Deep vein Doppler ultrasound of left lower extremity showed that common iliac vein common femoral vein, external iliac vein and superficial and deep femoral vein & popliteal vein were enlarged and contained with echogenous clot

Other parameters such as anti-thrombin 3, protein C&S and immunologic tests like ANA, Anti-dsDNA, antiPhospholipid antibodies (IgG) were within normal ranges. Erythrocyte sedimentation rate (ESR) was 28 mm/h. The D-dimer test was positive (6410.93 mg/L with normal value of <500 mg/L). Other parameters such as anti-thrombin 3, protein C&S and immunologic tests like ANA, Anti-dsDNA, antiPhospholipid antibodies (IgG) were within normal ranges. The tubal standard agglutination test (Wright test) and 2ME test were positive (in a titer of 1/1280 and 1/640, respectively). The chest x- ray and ECG were normal. Peripheral venous doppler ultrasound of left lower extremity showed that common iliac vein, common femoral vein, external iliac vein, superficial and deep femoral vein and popliteal vein enlarged and contained with echogenous clot. The clot had extended into the saphenofemoral valve. Anterior & posterior tibial veins also had no good flow and clots were seen. Marginal fine flow in common femoral vein was seen (figure 1). 

There was no effusion in sonographic evaluation of left hip joint. Subcutaneous soft tissue edema in left lower extremity was reported and few inguinal lymph nodes were detected on the same side. The above mentioned findings suggest the present of disseminated clot in the left lower extremity. The patient was diagnosed with thrombophlebitis due to brucellosis. He was advised to have bed rest and left leg elevation. He was treated with rifampicin 600mg once a day, doxycycline 100 mg twice a day (both for three months) and amikacin 500 mg twice a day (for 2 weeks). Also, low molecular weight heparin was started and after one week, it was altered with warfarin (with careful and frequent monitoring of PT & INR). Four days later, his leg pain and swelling decreased and he walked without any help. Ten days after the onset of this treatment, thrombophlebitis was cured. Warfarin was discontinued after six months. Follow up of the patient showed no abnormality after approximately one year later.

## Discussion

Brucellosis is an endemic disease in Iran and we usually see these cases as unusual form. Vascular infection is rarely seen during brucellosis. Among the cardiovascular infection, endocarditis is the most common presentation of cardiovascular involvement, which is reported in less than 2% of patients (2, 4). Deep vein thrombosis is a rare cardiovascular complication of brucellosis. 

The pathogenesis of this complication has not been described clearly but may be due to various reasons including granulomatous endophlebitis, induction of inflammation, the injury of perivascular tissue by the infection, the induction of a transient hypercoagulability or the immune reaction against brucellosis antigen (5). Several case reports with vascular involvement were reported in the medical literature.

Two cases with intracranial vascular thrombosis were reported in the medical literature (6, 7). Deep vein thrombosis in the left and right legs also were reported in the endemic regions (8, 9). 

In brucellosis endemic regions, clinicians who encounter patients with deep vein thrombosis with a current history of a febrile illness should consider the likelihood of brucellosis. A careful history, a meticulous physical examination and a rapid laboratory evaluation will assist the diagnosis.

## References

[1] Madkour MM, Cook GC, Zumla AI, Manson,s (2008). Brucellosis. tropical disease.

[2] Young EJ, Mondell GL, Bennett JE (2010). Brucella species. Principles and practice of Infectious diseases.

[3] Smailnejad Gangi SM, Hasanjani Roushan MR, Janmohammadi N (2012). Outcomes of treatment in 50 cases with spinal brucellosis in Babol, Northern Iran. J Infect Dev Ctries.

[4] Roushan MR, Amiri MJ (2013). Update on childhood brucellosis. Recent Pat Antiinfect Drug Discov.

[5] Corbel MJ (1997). Brucellosis: an overview. Emerg Infect Dis.

[6] Faraji F, Didgar F, Talaie-Zanjani A, Mohammadbeigi A (2013). Uncontrolled seizures resulting from cerebral venous sinus thrombosis complicating neurobrucellosis. J Neurosci Rural Pract.

[7] Zaidan R, Al Tahan AR (1999). Cerebral venous thrombosis: a new manifestation of neurobrucellosis. Clin Infect Dis.

[8] Koubaa M, Frigui M, Cherif Y (2013). Deep vein thrombosis associated with acute brucellosis: a case report and review of the literature. Korean J Intern Med.

[9] Odeh M, Pick N, Oliven A (2000). Deep venous thrombosis associated with acute brucellosis--a case report. Angiology.

